# The Risk of Virologic Failure Decreases with Duration of HIV Suppression, at Greater than 50% Adherence to Antiretroviral Therapy

**DOI:** 10.1371/journal.pone.0007196

**Published:** 2009-09-29

**Authors:** Michael Rosenblum, Steven G. Deeks, Mark van der Laan, David R. Bangsberg

**Affiliations:** 1 Department of Medicine, University of California San Francisco, San Francisco, California, United States of America; 2 Division of Biostatistics, School of Public Health, University of California, Berkeley, California, United States of America; 3 Massachusetts General Hospital Center for Global Health, Harvard Medical School, Harvard Initiative for Global Health, Boston, Massachusetts, United States of America; University of Cape Town, South Africa

## Abstract

**Background:**

We hypothesized that the percent adherence to antiretroviral therapy necessary to maintain HIV suppression would decrease with longer duration of viral suppression.

**Methodology:**

Eligible participants were identified from the REACH cohort of marginally housed HIV infected adults in San Francisco. Adherence to antiretroviral therapy was measured through pill counts obtained at unannounced visits by research staff to each participant's usual place of residence. Marginal structural models and targeted maximum likelihood estimation methodologies were used to determine the effect of adherence to antiretroviral therapy on the probability of virologic failure during early and late viral suppression.

**Principal Findings:**

A total of 221 subjects were studied (median age 44.1 years; median CD4+ T cell nadir 206 cells/mm^3^). Most subjects were taking the following types of antiretroviral regimens: non-nucleoside reverse transcriptase inhibitor based (37%), ritonavir boosted protease inhibitor based (28%), or unboosted protease inhibitor based (25%). Comparing the probability of failure just after achieving suppression vs. after 12 consecutive months of suppression, there was a statistically significant decrease in the probability of virologic failure for each range of adherence proportions we considered, as long as adherence was greater than 50%. The estimated risk difference, comparing the probability of virologic failure after 1 month vs. after 12 months of continuous viral suppression was 0.47 (95% CI 0.23–0.63) at 50–74% adherence, 0.29 (CI 0.03–0.50) at 75–89% adherence, and 0.36 (CI 0.23–0.48) at 90–100% adherence.

**Conclusions:**

The risk of virologic failure for adherence greater than 50% declines with longer duration of continuous suppression. While high adherence is required to maximize the probability of durable viral suppression, the range of adherence capable of sustaining viral suppression is wider after prolonged periods of viral suppression.

## Introduction

Medication adherence is the most important predictor of viral suppression among HIV infected patients receiving combination antiretroviral therapy [1]–[3]. The adherence threshold required to achieve durable viral suppression has declined with more potent regimens, such that the majority of patients in clinical practice are now able to maintain undetectable viral loads at adherence proportions as low as 70% [4]–[7]. Although the relationship between adherence and the short-term virologic response to therapy has been well described, the impact of successful treatment duration on the relationship between adherence and viral suppression remains unexamined.

Several independent observations suggest that the degree of drug pressure necessary to initially achieve viral suppression may be higher than that needed to maintain viral suppression. During effective antiretroviral therapy, plasma HIV RNA levels decline in a characteristic multi-phasic manner. The rapid first phase decay likely reflects the death of actively turning over, short-lived CD4+ T cells, while each subsequent phase likely reflects the death of longer lived cellular reservoirs [8]. After a period of 2 to 5 years, most patients reach a new steady-state in which the long-lived reservoir (presumed to be resting CD4+ T cells) continues to produce a steady-state level of viremia [9], [10]. Since the size of the reservoir containing replication competent virus declines over time [11], it is reasonable to postulate that the amount of virus that is able to initiate new rounds of replication also declines, as does the reservoir of pre-existing drug-resistant variants. Long-term HAART (highly active antiretroviral therapy) is also associated with decline in the number of activated and/or proliferating CD4+ T cells [12], [13]. Since these cells are the primary target cells for virus production, it is likely that the ability of virus to escape drug pressure declines proportionally with the decline in these cells [14]. These observations have been used as a rationale for a series of induction-maintenance clinical trials, in which patients are initially treated with more potent regimens and then later switched to a better tolerated, less potent regimen. Although many of the earlier trials failed [15], [16], more recent studies using longer induction periods and/or better maintenance regimens have provided some support for this approach [17], [18].

Based on these theoretical considerations, we hypothesized that the impact of adherence on the probability of virologic failure would differ depending on how long a subject had maintained virologic suppression. To test this hypothesis, we examined the effect of adherence on viral load after different durations of viral suppression. Subjects for this study were enrolled in a systematic community-based sample of HIV infected urban poor individuals living in San Francisco (the REACH cohort) [19]. Adherence was measured using unannounced pill counts at the participant's usual place of residence, as previously described [20]. This method has a close association with concurrent viral load [2], electronic pill cap adherence assessment [20], development of resistance [21], [22], and progression to AIDS [23]. We used marginal structural models [24]–[26] and targeted maximum likelihood estimation [27] to adjust for potential confounders of adherence and viral suppression.

## Methods

### Ethics statement

The University of California San Francisco and Committee on Human Subjects Research and the Partner's Human Research Committee approved all procedures.

### Study design and subject recruitment

Participants enrolled in the REACH cohort were invited to participate in a substudy focused on intensive adherence monitoring, as previously described [25], [28], [29]. Briefly, subjects had unannounced visits by research staff at their usual place of residence every three to six weeks, over a one year period. Percent adherence was determined from the number of remaining or unused antiretroviral pills and number of pills refilled between visits [2]. Three hundred and fifty seven HIV-positive subjects were monitored for medication adherence. The earliest monitoring period started in March 1998 and the latest monitoring date was in October 2007.

### Confounding variable assessment

Confounders of the effect of current adherence on virologic failure included the following: prior adherence, prior duration of HAART, prior exposure to mono/dual nucleoside therapy, recent CD4+ T cell count (lagged 2 months), CD4+ T cell nadir (lagged 2 months), demographics (sex, ethnicity, age), years of education, past and current antiretroviral treatment characteristics, crack cocaine and alcohol use, calendar time, and homelessness. Current antiretroviral treatment was classified as one of five potential regimens: ritonavir boosted protease inhibitor containing regimen, unboosted protease inhibitor containing regimen, non-nucleoside reverse transcriptase inhibitor containing regimen (NNRTI), combined non-nucleoside reverse transcriptase inhibitor-protease inhibitor containing regimen (PI/NNRTI), and nucleoside reverse transcriptase inhibitor (NRTI) only containing regimen.

### Specimen collection

Blood was drawn monthly for HIV RNA levels (viral loads) and quarterly for CD4+ T cell levels. HIV-1 viral load was performed using the HIV-1 Amplicor Monitor Version 1.0 ultra sensitive assay (Roche Molecular Systems, Alameda, CA).

### Statistical analyses

For every month of observation, each REACH cohort participant was classified as experiencing either virologic suppression (defined as HIV RNA less than 50 copies/ml) or virologic failure (defined as HIV RNA at least 50 copies/ml). Each participant in the REACH cohort who was suppressed for at least two consecutive months during adherence monitoring contributed data to the analysis; there were 221 participants who met this criterion. For each such participant, his/her data was included starting at the first month of viral suppression during adherence monitoring and continuing until virologic failure occurred or the adherence monitoring period ended.

Duration of suppression at any month was defined as the number of prior consecutive months of viral suppression. In calculating the duration of viral suppression, we used data on viral loads prior to the initiation of adherence monitoring.

We used marginal structural models to estimate the effect of adherence during a given month on the probability of virologic failure at the end of that month. These effects were estimated within subpopulations defined by duration of prior viral suppression. For example, among participants who had maintained viral suppression for exactly 4 consecutive months, we estimated the effect of adherence during month 5 on the probability of virologic failure at the end of month 5. For our primary analysis, adherence was stratified into the following four categories: 0−49%, 50−74%, 75−89%, or 90−100% pills taken. The marginal structural model was fit using a targeted maximum likelihood estimator (TMLE) [27], in order to adjust for the potential confounders listed above. The TMLE, in this application, relies on 1) a multinomial logistic regression model for predicting medication adherence given potential confounders and number of consecutive months virally suppressed and 2) a logistic regression model for the probability of virologic failure given adherence, confounders, and number of consecutive months virally suppressed. The TMLE is robust to model misspecification, in that it gives asymptotically unbiased estimates whenever at least one of the models (1) or (2) is correct. It also has several advantages over alternative estimation techniques, such as inverse probability of treatment weighting [30], g-computation [31], [32], and doubly robust estimators [33]–[36]; these advantages have been previously described [27].

Based on the marginal structural model fit using targeted maximum likelihood estimation, we estimated the probabilities of virologic failure at each set stratum of adherence, for duration of continuous suppression ranging from 1 to 12 months. Confidence intervals for the difference between the probability of failure after 1 month of continuous suppression and the probability of failure after 12 months of continuous suppression, at each adherence stratum, were calculated using 10,000 iterations of the nonparametric bootstrap (BCa method) [37].

We tested for effect modification based on the estimated coefficients of the marginal structural models. More precisely, we tested whether the effect of the proportion of medication taken on the probability of virologic failure in the current month, is impacted by the number of consecutive months one has been virally suppressed. This test involved first computing the logarithm of the causal relative risk of virologic failure, comparing high adherence (90–100%) vs. low adherence (0–49%), conditional on number of consecutive months virally suppressed. This causal relative risk was based on the fit of the marginal structural model discussed above. Next, we used a linear regression model to test the null hypothesis that the causal relative risk does not depend on the number of consecutive months virally suppressed. Standard errors were computed using the nonparametric bootstrap as above.

Time-lagged confounder measurements were used to ensure that confounders occurred before, and thus could not be influenced by, medication adherence. We imputed missing confounder values by carrying the most recent observation forward. Because subjects could contribute data at multiple time points, bootstrap resampling was based on subject rather than data-point.

## Results

### Participant characteristics

Of the 357 subjects in the REACH cohort who received unannounced pill counts, 221 met our criteria for inclusion in the analysis. The median age at start of adherence monitoring was 44.1 years (IQR 10.5). The median CD4+ T cell count was 390 cells/mm^3^ and the median CD4+ T cell nadir was 206 cells/mm^3^. Fifty-six of the 221 participants (25%) were receiving an unboosted protease inhibitor based regimen. The mean observed duration of continuous viral suppression was 6.7 months. Of the 1201 subject-months of observation, 95 (8%) were missing adherence measurements and 159 (13%) were missing viral load measurements. See Tables 1 and 2 for a more extensive list of participant characteristics.

**Table 1:** Participant Characteristics at Start of Adherence Monitoring.

Characteristic	Among Subjects Achieving Viral Suppression During Adherence Monitoring (n = 221)	Missing (%)
Non-Caucasian (%)	130 (59%)	3 (1%)
Male (%)	149 (67%)	10 (5%)
Median age (IQR)	44.1 (10.5)	0
Antiretroviral Treatment		
PIbased (%)	56 (25%)	0
NNRTI -based (%)	81 (37%)	0
PI-NNRTI-based (%)	15 (7%)	0
NRTI-only (%)	7 (3%)	0
Ritonavir boosted protease inhibitor based	62 (28%)	0
Once daily therapy (%)	81 (37%)	0
Median months on current regimen (IQR)	7 (13)	0
Median number of ARV regimens experienced (IQR)	2 (2)	2 (<1%)
ARV naïve (%)	100 (46%)	2 (<1%)
Mono or dual nucleoside exposure (%)	81 (37%)	2 (<1%)

**Table 2:** Participant Characteristics During Follow-up.

Characteristic	Among Subjects Achieving Viral Suppression (n = 221)	Missing (%)
Intravenous drug use reported in last 30 days at least once during follow-up (%)	34 (15%)	31 (14%)
Crack use (%)	44 (20%)	31 (14%)
Slept on street or in shelter (%)	12 (5%)	36 (16%)
Mean days intoxicated in past month (SD)	3.5 (7.2)	31 (14%)
Median nadir CD4+/ml (IQR)	206 (279)	30 (14%)
Median CD4+/ml (IQR)	390 (338)	5 (2%)
Person-months with pill box organizer use, excluding subject-months with missing data (%)	274 (45%)	0 (0%)
Mean duration of continuous viral suppression in months (SD)	6.7 (4.3)	0 (0%)
Mean viral load at month of failure, in log copies/ml (IQR)	2.7 (1.0)	0 (0%)
Median pill count adherence (IQR)	0.92 (0.25)	95 (8%)

### Adherence over time

Unannounced pill count adherence ranged from 0 to 100%, with a median of 92%. In univariate analyses, higher adherence was associated with higher past adherence, longer duration of viral suppression, higher CD4+ nadir, and white/Caucasian ethnicity, while lower adherence was associated with use of crack, intravenous drug use, and Black/African American ethnicity. Comparing observed adherence proportions among subjects who maintained viral suppression for different durations of time, there was a gradual decrease in the proportion with 0–49% adherence (from 11% of subject-months at 1 month of suppression to 5% of subject-months at 12 months of continuous suppression) and 50–74% adherence (from 19% of subject-months at 1 month of suppression to 15% of subject-months at 12 months of continuous suppression). There was a corresponding gradual increase in the proportion with 75–89% adherence (from 19% of subject-months at 1 month of suppression to 25% of subject-months at 12 months of continuous suppression) and 90–100% adherence (from 50% of subject-months at 1 month of suppression to 55% of subject-months at 12 months of continuous suppression). None of these changes in the proportion of participants at a given adherence level was statistically significant at the 0.05 level.

### Duration of viral suppression and incidence of virologic failure

Of the 221 participants who achieved viral suppression during adherence monitoring, 108 (49%) subsequently experienced virologic failure during adherence monitoring. Based on univariate regression analyses, participants who had longer duration of suppression also had higher CD4+ counts, higher CD4+ nadir, and higher cumulative average adherence proportions. Participants who had longer duration of suppression were also more likely to be on an NNRTI or PI/NNRTI regimen than an unboosted protease inhibitor regimen. Virologic failure (defined as a plasma HIV RNA greater than 50 copies/ml) was associated with low recent CD4+ T cell count, low past adherence, shorter duration of prior suppression, and intravenous drug use.

A logistic regression of the probability of virologic failure on adherence, confounders, and number of months since initial suppression was used in the targeted maximum likelihood analysis (see Table 3). Based on this logistic regression, a lower risk of virologic failure was associated with longer duration of continuous suppression, lower past viral load, and being on an NNRTI-based regimen.

**Table 3:** Multivariate Regression of Virologic Failure on Adherence, Duration of Continuous Suppression, and Confounders.

Term in Multivariate Linear Regression Model	Coefficient	95% CI Lower Limit	95% CI Upper Limit
Indicator of Adherence 0–49%	0.51	−1.02	1.76
Indicator of Adherence 50–74%	0.72	−0.48	1.76
Indicator of Adherence 75–89%	−0.06	−1.44	1.1
Months of Continuous Suppression	−0.27	−0.53	−0.11
Indicator of Interaction: Adherence 0–49% x Months of Continuous Suppression	0.24	−0.03	0.54
Indicator of Interaction: Adherence 50–74% x Months of Continuous Suppression	0.04	−0.24	0.33
Indicator of Interaction: Adherence 75–89% x Months of Continuous Suppression	0.18	−0.09	0.46
Once daily therapy	0.29	−0.61	1.07
Pillbox Use	0.33	−0.59	1.19
CD4 T cell count (2 months prior) (per 100 cells)	−0.01	−0.01	0
Nadir CD4 T cell count (2 months prior) (per 100 cells)	0	−0.01	0
Viral load (2 months prior) (per 100,000 copies)	0.57	0.13	1.06
Calendar month (per 30 days)	−0.01	−0.03	0
Months on current regimen (per 30 days)	−0.01	−0.02	0
Age (per year)	−0.01	−0.05	0.03
Number of days intoxicated (in past month)	−0.02	−0.07	0.02
Intravenous drug use	0.39	−0.6	1.27
Slept on street or in shelter	1.21	−0.59	2.49
Crack use	0.2	−0.82	1.01
Man	0.17	−0.61	0.9
Black/African-American (Ethnicity Response “Other” used as baseline)	0.95	−0.04	1.88
Hispanic/Latino	0.71	−0.96	2.06
White/Causasian	0.58	−0.48	1.46
Mono or dual nucleoside exposure	0.58	−0.35	1.35
Unboosted PI-based regimen	0.05	−0.93	1
NNRTI-based regimen	−0.99	−1.68	−0.07
PI-NNRTI-based regimen	−1.24	−2.88	0.37
NRTI only regimen	−0.13	−4.14	2.21
Number of regimens experienced	−0.17	−0.35	0.04
Depression (BDI>14)	0.61	−0.31	1.29
Years of Education	0.08	−0.06	0.23
Adherence lagged 1 month	−0.02	−1.97	2.27
Cumulative mean adherence lagged 1 month	0.71	−2.33	3.5

### Duration of viral suppression modifies the effect of adherence on virologic failure

**Figure 1:**
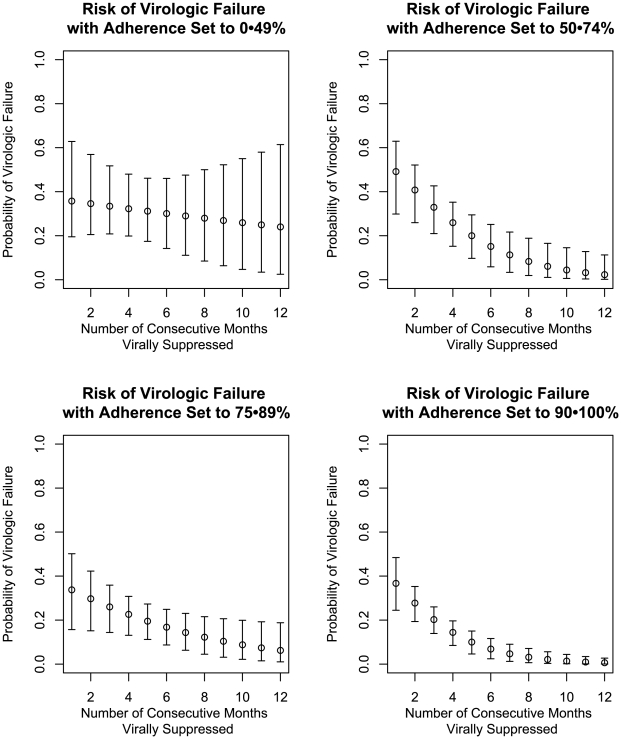
Estimates and 95% Confidence Intervals for the Risk of Virologic Failure, at Four Ranges of Adherence, Given Duration of Continuous Viral Suppression.

We first estimated the causal effect of different adherence proportions on the probability of virologic failure, conditioned on duration of continuous viral suppression (Figure 1). We stratified subject-months by corresponding duration of suppression and then estimated the probability of virologic failure setting the adherence proportion to be in the ranges (0–49%, 50–74%, 75–89%, and 90–100%). Because adherence proportion cannot be randomly assigned as an intervention, we relied on a marginal structural model and targeted maximum likelihood estimation of the causal effects of adherence on viral suppression at each time point.

At one month of suppression, the estimated probabilities of virologic failure at all four adherence categories were relatively high: 0.36 for 0–49% adherence, 0.49 for 50–74% adherence, 0.34 for 75–89% adherence, and 0.37 for 90–100%. The widths of the confidence intervals for these estimates were large (ranging from 0.24 to 0.43), and there was a substantial region of mutual overlap among all four confidence intervals.

The point estimates for the probability of virologic failure decreased with longer duration of suppression (Figure 1). The probabilities of virologic failure after 12 months of viral suppression were 0.24, 0.02, 0.06, and 0.01 for adherence categories of 0–49%, 50–74%, 75–89%, and 90–100%, respectively. Comparing the probability of failure just after achieving suppression versus after 12 consecutive months of suppression, there was a statistically significant decrease in the probability of virologic failure for adherence set above 50%. The estimated risk difference, comparing the probability of virologic failure after 1 month vs. after 12 months of continuous viral suppression was 0.47 (95% CI 0.23–0.63) for the 50–74% adherence stratum, 0.29 (95% CI 0.03–0.50) for 75–90% adherence stratum, and 0.36 (95% CI 0.23–0.48) for the 90–100% adherence stratum. The estimated risk of failure decreased by only 0.13 (95% CI −0.74–0.39) for the 0–49% adherence stratum, which was not statistically significant at the 0.05 level.

We carried out a test for effect modification by number of consecutive months virally suppressed. We considered the causal relative risk, comparing the effect of different adherence proportions on the probability of virologic failure. This causal relative risk was calculated conditioning on the number of months of viral suppression, using a marginal structural model. The null hypothesis that this causal relative risk does not depend on the number of consecutive months virally suppressed was rejected (p-value 0.001). Thus, the data provide evidence for duration of suppression being an effect modifier of the adherence-suppression relationship.

## Discussion

Treatment adherence is widely accepted as the primary determinant of long-term virologic outcomes among antiretroviral-treated patients. These data suggest that for adherence proportions greater than 50%, the probability of virologic failure decreases with longer duration of viral suppression. For example, we estimated the risk of virologic failure for adherence between 75–89% (which is the most common adherence range in most chronic diseases) to be 0.31 after 1 month of suppression. In contrast, we estimated the risk of virologic failure for adherence between 75–89% to be 0.06 after 12 months of suppression. Similar trends were observed in those with adherence in the ranges 50 to 74%, and 90 to 100%.

Consistent with our finding that the impact of adherence on viral suppression is modified by history of successful treatment are a number recent induction-maintenance studies. For example, lopinavir-ritonavir monotherapy leads to higher rates of viral suppression once patients achieve suppression on standard regimens than when lopinvavir-ritonavir monotherapy is used as de novo [38], [39]. Similarly, the use of a triple nucleoside reverse transcriptase inhibitor appears to be more effective after viral suppression is achieved than when the regimen is used as an initial regimen [17], [40].

One important limitation in our analysis is the potential role of selection bias in explaining the decrease in probability of virologic failure among those with longer duration of suppression. That is, those susceptible to virologic failure may have been selected out at earlier months, leaving a group with lower probability of failure at later months. Thus, we cannot exclude selection bias as a potential explanation for these findings.

There are several other limitations that deserve comment. While we did have extensive and systematic adherence data, we did not have as extensive virologic suppression data on all individuals, since some people entered the cohort already on treatment. These individuals may have had additional periods of viral suppression that were not included in this analysis. The estimation method we used relied on having included all confounders of adherence and virologic failure in our analyses, and on our marginal structural model and other models used being correctly specified. While we included many of the known predictors of adherence and virologic failure, unmeasured confounders may lead to bias in our estimates. Finally we studied patients on a wide range of antiretroviral therapy and some regimens we studied are no longer commonly used. We did not have the statistical power to limit analyses to just individuals on NNRTI or ritonavir boosted protease inhibitor based regimens.

While the adherence proportion required to sustain viral suppression may decline over time, the goal of near perfect adherence should remain unchanged. While both more potent therapy and sustained viral suppression may lessen the virologic consequences of missed doses, improving adherence will increase the probability of durable and sustained viral suppression [7], [41].
